# Cortical lesions, central vein sign, and paramagnetic rim lesions in multiple sclerosis: Emerging machine learning techniques and future avenues

**DOI:** 10.1016/j.nicl.2022.103205

**Published:** 2022-09-24

**Authors:** Francesco La Rosa, Maxence Wynen, Omar Al-Louzi, Erin S Beck, Till Huelnhagen, Pietro Maggi, Jean-Philippe Thiran, Tobias Kober, Russell T Shinohara, Pascal Sati, Daniel S Reich, Cristina Granziera, Martina Absinta, Meritxell Bach Cuadra

**Affiliations:** a Signal Processing Laboratory (LTS5), Ecole Polytechnique Fédérale de Lausanne (EPFL), Lausanne, Switzerland; b CIBM Center for Biomedical Imaging, Switzerland; c Department of Neurology, Icahn School of Medicine at Mount Sinai, New York, NY, USA; d ICTeam, UCLouvain, Louvain-la-Neuve, Belgium; e Louvain Inflammation Imaging Lab (NIL), Institute of Neuroscience (IoNS), UCLouvain, Brussels, Belgium; f Radiology Department, Lausanne University and University Hospital, Switzerland; g Translational Neuroradiology Section, National Institute of Neurological Disorders and Stroke, National Institutes of Health, Bethesda, MD, USA; h Department of Neurology, Cedars-Sinai Medical Center, Los Angeles, CA, USA; i Advanced Clinical Imaging Technology, Siemens Healthcare AG, Lausanne, Switzerland; j Department of Neurology, Cliniques universitaires Saint-Luc, Université catholique de Louvain, Brussels, Belgium; k Department of Neurology, CHUV, Lausanne, Switzerland; l Center for Biomedical Image Computing and Analysis (CBICA), Department of Radiology, University of Pennsylvania, Philadelphia, PA, USA; m Penn Statistics in Imaging and Visualization Endeavor (PennSIVE), Center for Clinical Epidemiology and Biostatistics, University of Pennsylvania, Philadelphia, PA, USA; n Department of Biostatistics, Epidemiology, and Informatics, Perelman School of Medicine, University of Pennsylvania, Philadelphia, PA, USA; o Translational Imaging in Neurology (ThINk) Basel, Department of Biomedical Engineering, Faculty of Medicine, University Hospital Basel and University of Basel, Switzerland; p Neurologic Clinic and Policlinic, MS Center and Research Center for Clinical Neuroimmunology and Neuroscience Basel (RC2NB), University Hospital Basel and University of Basel, Basel, Switzerland; q IRCCS San Raffaele Hospital and Vita-Salute San Raffaele University, Milan, Italy; r Department of Neurology, Johns Hopkins University School of Medicine, Baltimore, MD, USA

**Keywords:** MS, multiple sclerosis, MRI, magnetic resonance imaging, DL, deep learning, ML, machine learning, CL, cortical lesions, PRL, paramagnetic rim lesions, CVS, central vein sign, WML, white matter lesions, FLAIR, fluid-attenuated inversion recovery, MPRAGE, magnetization prepared rapid gradient-echo, GM, gray matter, WM, white matter, PSIR, phase-sensitive inversion recovery, DIR, double inversion recovery, MP2RAGE, magnetization-prepared 2 rapid gradient echoes, SELs, Slowly evolving/expanding lesions, CNN, convolutional neural network, XAI, explainable AI, PV, partial volume

## Abstract

The current diagnostic criteria for multiple sclerosis (MS) lack specificity, and this may lead to misdiagnosis, which remains an issue in present-day clinical practice. In addition, conventional biomarkers only moderately correlate with MS disease progression. Recently, some MS lesional imaging biomarkers such as cortical lesions (CL), the central vein sign (CVS), and paramagnetic rim lesions (PRL), visible in specialized magnetic resonance imaging (MRI) sequences, have shown higher specificity in differential diagnosis. Moreover, studies have shown that CL and PRL are potential prognostic biomarkers, the former correlating with cognitive impairments and the latter with early disability progression. As machine learning-based methods have achieved extraordinary performance in the assessment of conventional imaging biomarkers, such as white matter lesion segmentation, several automated or semi-automated methods have been proposed as well for CL, PRL, and CVS. In the present review, we first introduce these MS biomarkers and their imaging methods. Subsequently, we describe the corresponding machine learning-based methods that were proposed to tackle these clinical questions, putting them into context with respect to the challenges they are facing, including non-standardized MRI protocols, limited datasets, and moderate inter-rater variability. We conclude by presenting the current limitations that prevent their broader deployment and suggesting future research directions.

## Highlights

• Cortical lesions, paramagnetic rim lesions, and the central vein sign are emerging imaging biomarkers in MS.
• Their assessment requires advanced MRI techniques and high expertise.
• We discuss machine learning techniques proposed to automatically assess them.
• The methods’ current limitations and future research directions are presented.

## 1 Introduction

Multiple sclerosis (MS) is a chronic inflammatory disease and a common cause of neurological disability in young adults (Reich et al., 2018). Its hallmark is demyelinated white matter lesions (WML) forming in the central nervous system (Reich et al., 2018). These lesions are assessed in-vivo with magnetic resonance imaging (MRI), which is the imaging technique of choice to diagnose MS and monitor the disease over time (Hemond and Bakshi, 2018). The current MRI diagnostic criteria (McDonald criteria) are based on the dissemination in space and time of such lesions (Thompson et al., 2018). Moreover, the quantification of the total lesion volume is important to determine ongoing disease activity and monitor treatment effect over time (Giorgio et al., 2014). Recommended MRI techniques include T2 and T1-weighted inversion recovery sequences, such as fluid-attenuated inversion recovery (FLAIR), and magnetization prepared rapid gradient-echo (MPRAGE) (Wattjes et al., 2021). At common clinical magnetic fields (1.5 T and 3 T), the use of gadolinium-based contrast agents is useful to evaluate patients suspected of MS and monitor disease activity causing breakdown of the blood–brain barrier (Filippi et al., 2019).

As the manual detection of WML is time-consuming and prone to inter-rater variability (Hagens et al., 2019), a myriad of automated or semi-automated approaches have been developed to facilitate this task (Lladó et al., 2012). These methods were initially based primarily on MRI intensity features and probabilistic atlases (Lladó et al., 2012), whereas, more recently, the vast majority use deep learning (DL) approaches (Zeng et al., 2020), without prior feature extraction. Substantial effort is now being made towards reproducibility of the results and open science (Vrenken et al., 2021). Several grand challenges have been organized (Carass et al., 2017, Commowick et al., 2018, Commowick et al., 2021), in which DL-based methods have achieved the best performance, approaching or sometimes even outperforming human readers (Carass et al., 2017, Commowick et al., 2021). WML segmentation methods have been reviewed recently (Zeng et al., 2020, Kaur et al., 2021); the present review thus focuses on machine learning techniques tailored for lesional biomarkers specific to MS that require advanced MRI techniques and have the potential to improve MS diagnosis and prognosis.

One major drawback of the current MS diagnostic criteria is their lack of specificity, as they were proposed to identify patients with a high likelihood of MS rather than distinguish MS from other conditions (Thompson et al., 2018). The lack of specificity of these criteria may lead to misdiagnosis, which remains a persistent problem of MS (Solomon et al., 2019). Multi-center studies have shown a misdiagnosis rate of 18% (Kaisey et al., 2019), often associated with atypical clinical or imaging findings. Improving the diagnostic specificity would prevent harmful consequences for patients (Solomon et al., 2016) and allow clinicians to prescribe the appropriate treatment earlier. In addition, although clinical relapses are often associated with the appearance of new WML, the overall WML burden, which is the most common MRI biomarker examined in clinical routine, is only moderately correlated with disability and poorly predicts transition to progressive disease (Barkhof, 2002). For all these reasons, there is a need for additional biomarkers that are highly specific to MS or correlate with disease progression.

Quantitative MRI, such as relaxometry, myelin imaging, or diffusion MR, provides information related to the microstructural composition and organization of tissues. In MS, quantitative MRI techniques complement conventional MRI techniques by providing insights into disease mechanisms (Granziera et al., 2021). For instance, diffusion tensor imaging and microstructure models of diffusion can help better understand the MS lesion heterogeneity (myelin and axonal damage). Voxel-wise analysis methods allow exploring group-wise differences without the need for prior lesion segmentation (Thaler et al., 2021;16(2):e0245844.). On the contrary, classification methods in this context have been used to cluster different lesion types based on prior lesion segmentation and derived scalar measurements from diffusion-based measurements (FA, MD, NODDI parameters, etc) averaged at the lesion level (Lu et al., 2021, Oladosu et al., 2021, Ye et al., 2020, Martínez-Heras et al., 2020). Further studies, however, are still needed to verify the possible use of these quantitative features for patient stratification.

Recently, advances in MR technology, such as the development of specialized sequences, acceleration of protocols, and the proliferation of ultra-high field MRI, have allowed the imaging of pathologically specific MS lesional biomarkers (Cortese et al., 2019, Ineichen et al., 2021). These include cortical lesions (CL), the central vein sign (CVS), and paramagnetic rim lesions (PRL). Studies have shown that CL and PRL are potential prognostic biomarkers: CL are associated with cognitive impairments, while patients with PRL experience an earlier progression in disability (Calabrese et al., 2010, Absinta et al., 2019). Furthermore, the CVS and PRL have proven to be effective for differentiating MS from mimicking diseases (Ontaneda et al., 2021, Sati et al., 2016, Clarke et al., 2020, Maggi et al., 2020). All three biomarkers, however, require dedicated MRI sequences at high (3 T) or ultra-high (7 T) magnetic fields, and experienced raters for their manual assessment, which can be very time-consuming. As done in the past for WML, various automated or semi-automated methods, mostly based on machine learning (ML), have been developed to facilitate the three aforementioned biomarkers’ assessment (see Table 1). Compared to their WML counterparts, however, they face additional challenges, including non-standardized imaging protocols, moderate inter-rater variability when determining ground truth annotations, and smaller datasets. Automated assessment could improve standardization and facilitate large-scale assessment in clinical routine of the aforementioned biomarkers, with clear benefits in terms of MS diagnosis and prognosis.

**Table 1:** Summary of the methods proposed for the automated or semi-automated analysis of cortical lesions, the central vein sign, and paramagnetic rim lesions. The task is abbreviated as follows: segmentation (S), classification (C). If not specified, all sequences were 3D. Other abbreviations: k-nearest neighbors algorithm (K-NN), convolutional neural network (CNN), partial volume (PV).

Biomarker	Authors (year)	Method	Task	MRI sequences (magnetic field strength)	Dataset size (n. of sites)	Code available
Cortical lesions	Tardif,C. L., et al. (Tardif et al., 2010) (2010)	Laminar profile shape analysis	S	Quantitative high-resolution scan (3 T)	1 post mortem brain scan (1)	No
	Fartaria, M.J., et al. (Fartaria et al., 2016) (2016)	K-NN	S	FLAIR, MPRAGE, MP2RAGE, DIR (3 T)	39 MS patients (1)	No
	Fartaria, M.J., et al. (Fartaria et al., 2017) (2017)	K-NN with partial volume constraints	S	FLAIR, MPRAGE, MP2RAGE, DIR (3 T)	39 MS patients (1)	No
	Fartaria, M.J., et al. (Fartaria et al., 2019)(2019)	PV estimation and topological constraints	S	MP2RAGE (7 T)	25 MS patients (2)	No

	La Rosa, F., et al. (La Rosa et al., 2020) (2020)	CNN	S	FLAIR, MP2RAGE (3T)	90 MS patients (2)	Yes^b^
	La Rosa, F., et al. (La Rosa et al., 2020) (2020)	CNN	S and C	MP2RAGE, 2D T2*-w GRE, T2*w 3D-*EPI* (7T)	60 MS patients (1)	Yes^c^
	La Rosa, F., et al (2022) (La Rosa et al., 2022)	CNN	S and C	MP2RAGE, 2D T2*-w GRE, T2*w 3D-*EPI* (7T)	80 MS patients (2)	Yes^c^
Paramagnetic rim lesions	Barquero, G., et al. (Barquero et al., 2020) (2020)	CNN	C	FLAIR, T2*w 3D-*EPI* (3T)	124 MS patients (2)	No
	Lou, C., et al. (Lou et al., 2021) (2021)	Random forest classifier	C	FLAIR, T1-w, T2*w 3D-*EPI* (3T)	20 MS patients (1)	Yes^d^
	Zhang, H. et al. (2022) (Zhang et al., 2022)	Residual network and radiomic features	C	FLAIR, QSM (3T)	172 MS patients (1)	No
Central vein sign	Maggi, P., Fartaria, MJ, et al. (Maggi et al., 2020) (2020)	CNN	C	T2*w 3D-*EPI*, FLAIR (3T)	42 MS patients, 33 mimics, 5 others (3)	No
	Dworkin, J. D., et al. (Dworkin et al., 2018) (2018)	Probabilistic method	C	T2*w 3D-*EPI*, FLAIR (3T)	16 MS patients, 15 MS mimics (1)	Yes^e^

^b^https://github.com/FrancescoLR/MS-lesion-segmentation

^c^https://github.com/Medical-Image-Analysis-Laboratory/CLaiMS

^d^https://github.com/carolynlou/prlr

^e^https://github.com/jdwor/cvs

In this review, we first briefly describe these advanced imaging biomarkers and their imaging requirements and then focus on image processing techniques tailored for their automated segmentation and classification. We conclude with a discussion on current limitations and future lines of research to boost the development of ML approaches in this area and encourage their adoption in MS research and clinical settings.

## 2 Cortical lesions, paramagnetic lesions, and central vein sign

In this section, we present a brief description of CL, CVS, and PRL, and their respective imaging protocols. In addition to the CVS and PRL, which have emerged as promising MS biomarkers in recent years, we also included CL which, although included in the MS diagnostic criteria, are not yet commonly analyzed in clinical practice. For the sake of completeness, a short description of slowly expanding lesions (SELs) is also provided, although these have not been assessed with ML-based approaches yet.

**Fig. 1:**
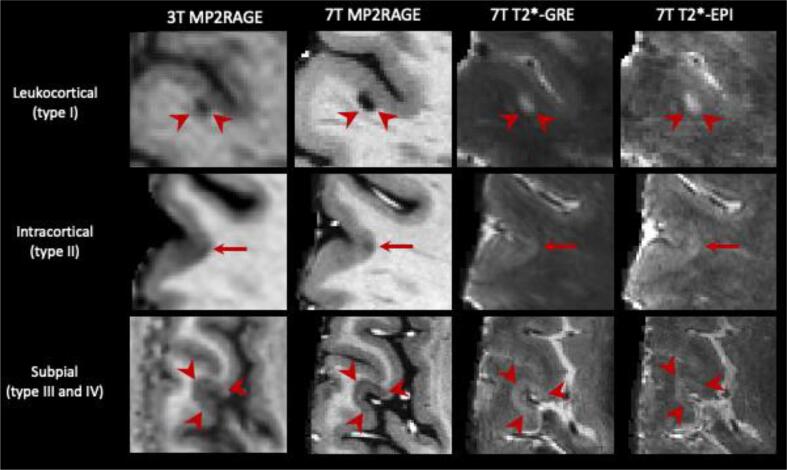
Representative examples of the three main types of CL. From left to right: 3 T MP2RAGE (0.75 mm isometric), 7 T MP2RAGE (0.5 mm isometric), 7 T T2*-*EPI* (0.5 mm isometric) and 7 T T2*-GRE (0.5 mm isometric). CL, including leukocortical, intracortical, and subpial subtypes, are seen better at 7 T due to higher signal-to-noise ratios, allowing higher resolution scans, and increased susceptibility effects. The 7 T MP2RAGE image shown was obtained as the average of 4 acquisitions.

**Fig. 2:**
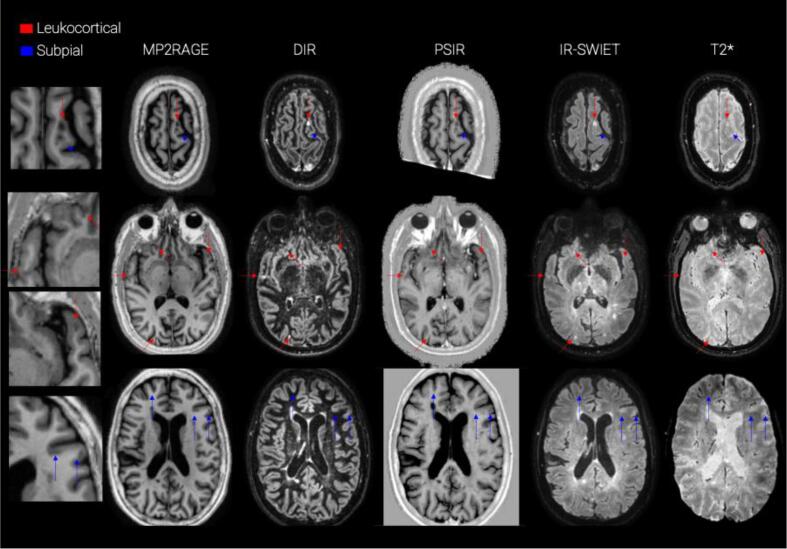
examples of CL seen in different MRI contrasts at 3 T. From left to right: MP2RAGE, DIR, PSIR, IR-SWIET, T2*. Red arrows point to leukocortical lesions and blue arrows to subpial lesions. (For interpretation of the references to colour in this figure legend, the reader is referred to the web version of this article.)

***Cortical lesions (CL) -*** Cortical lesions are a type of MS lesions that involve, at least partially, the cortex and have been classified into three main categories (Calabrese et al., 2010) (see Fig. 1): leukocortical lesions are located at the interface between WM and gray matter (GM) (type I), intracortical lesions are purely in the cortex and do not reach the pial surface (type II), and subpial lesions touch the subpial surface of the brain (type III) and sometimes extend all the way to the white matter (type IV). Cortical demyelination in MS has long been recognized in pathology studies, but only in the last two decades have dedicated sequences on high- and ultra-high field scanners provided in-vivo evidence of cortical damage (Calabrese et al., 2010). Cortical lesions are clinically interestingfor several reasons. First, they have been observed in the early stages of the disease and in all of the major MS phenotypes (Kidd et al., 1999). Second, they are associated with disability (Harrison et al., 2015, Nielsen et al., 2013, Calabrese et al., 2012) and in some studies, their number was associated with cognitive disability more strongly than the number of WML (Harrison et al., 2015, Favaretto et al., 2016). Third, longitudinal studies have linked them with disease progression (Treaba et al., 2019, Mainero et al., 2015, Scalfari et al., 2018, Calabrese et al., 2013). Fourth, subpial cortical demyelination is highly specific to MS (Junker et al., 2020); CL have been observed in patients with radiologically isolated syndrome (Giorgio et al., 2011), but not in patients with neuromyelitis optica (Sinnecker et al., 2012). Since 2017, CL have been included in the MS diagnostic criteria (Thompson et al., 2018), but their visualization from routine MRI sequences remains difficult. For instance, a postmortem study showed that 3D FLAIR at 3 T could detect about 41% of leukocortical lesions and only 5% of intracortical and subpial lesions (Geurts et al., 2005). This supports the need for specialized MRI techniques (see Fig. 2) such as the phase-sensitive inversion recovery (PSIR), double inversion recovery (DIR), and magnetization-prepared 2 rapid gradient echoes (MP2RAGE) (Filippi et al., 2019, Müller et al., 2022). However, these sequences are still relatively insensitive to CL at 1.5 T and 3 T (Müller et al., 2022, Kilsdonk et al., 2016, Beck et al., 2020). Ultra-high field MRI, with its higher signal-to-noise ratio and increased susceptibility effects, has proven to be a powerful tool for increasing the sensitivity to CL, especially for intracortical and subpial lesions (Madsen et al., 2021, Beck et al., 2022, Maranzano et al., 2019). Even with the most sensitive methods, however, CL are small and often subtle, making manual segmentation extremely time consuming and subject to relatively low inter-rater reliability (Harrison et al., 2015, Faizy et al., 2017;12(2):e0172923.).

**Fig. 3:**
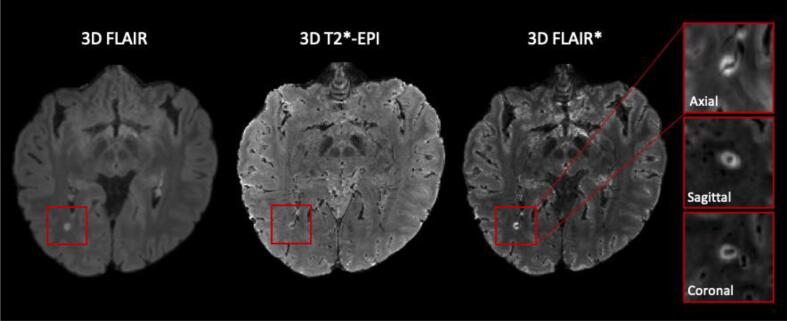
A central vein running through a lesion visible in the three planes (zoomed-in boxes) in a 3D FLAIR* obtained combining FLAIR and T2*-*EPI* acquisitions at 3 T. Resampling was applied to the magnified images for visualization purposes. FLAIR, T2*-*EPI* and FLAIR* are the MRI contrasts that have been used by ML approaches for CVS detection. Refer to the supplementary material for additional examples of the CVS on different susceptibility-weighted imaging sequences.

***Central vein sign (CVS) -*** Recently, studies have suggested that an MRI-detectable central vein inside MS lesions might be evidence of pathological processes specific to MS (see Fig. 3) (Maggi et al., 2018, Solomon et al., 2016). This marker, referred to as the “central vein sign,” has gained attention in recent years, as it could help to differentiate MS from mimicking diseases (Sati et al., 2016, Sinnecker et al., 2019, Ciotti et al., 2022, Sparacia et al., 2018, Tranfa et al., 2022). Small cerebral veins can be detected with susceptibility-based MRI sequences, taking advantage of the magnetic properties of venous blood that is rich in deoxyhemoglobin (Haacke et al., 2009, Mittal et al., 2009). The CVS can be reliably observed across different T2* sequences at 3 T, although the sensitivity depends on the sequence considered (Samaraweera et al., 2017). To obtain the best detection sensitivity for the CVS, optimized MRI acquisitions have been proposed (T2*-weighted acquired with 3D-segmented echo-planar-imaging or T2*w 3D-*EPI* (Sati et al., 2014), combined T2-FLAIR and T2*, also called FLAIR* (Sati et al., 2012), and susceptibility-based sequence, called SWAN-Venule (Gaitán et al., 2020). These sequences were shown to provide superior CVS detection compared to clinical acquisitions at 1.5 T and 3 T (Castellaro et al., 2020, Suh et al., 2019). Single-center and multi-center retrospective studies imaging patients with clinically established diagnoses have demonstrated a significantly higher proportion of CVS-positive white matter lesions (%CVS + ) in MS (mean pooled incidence: 79%, 95% CI: 68–87%) (Suh et al., 2019) as compared to other neurological disorders mimicking MS (mean pooled incidence: 38%, 95% CI: 18–63%) (Suh et al., 2019) such as cerebral small vessel disease (Campion et al., 2017), neuromyelitis optica spectrum disorder (NMOSD) (Cortese et al., 2018), inflammatory vasculopathies (Maggi et al., 2018), and migraine (Solomon et al., 2016). To distinguish MS from other neurological conditions, different CVS-based criteria have been proposed to date, some based on the percentage of perivenular lesions (from 35% to 60%) and others simply on the CVS lesion count (3-lesion or 6-lesion rule) (Maggi et al., 2018, Tallantyre et al., 2011, Mistry et al., 2016, Solomon et al., 2018). From a diagnostic perspective, retrospective studies have shown excellent diagnostic discrimination by applying the ‘40% rule’ (Tallantyre et al., 2011) with sensitivity = 91% [95% CI, 82%-97%] and specificity = 96% [95% CI, 88%-100%]) (Castellaro et al., 2020). However, applying percentage-based criteria requires manual exclusion of lesions that are confluent or have multiple or eccentric veins, and performing the CVS evaluation on all the remaining lesions present in patients’ brains, which is a time-consuming process difficult to accomplish in clinical practice.

**Fig. 4:**
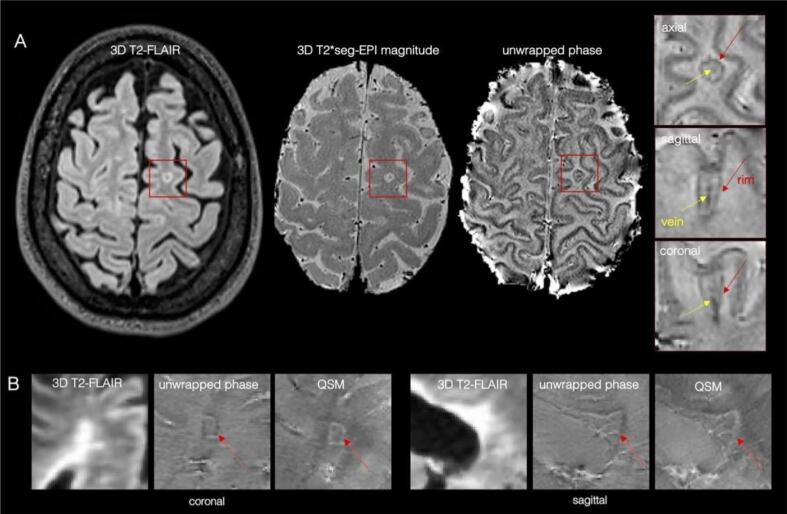
(A) Representative paramagnetic rim lesion seen on a 3 T T2*-weighted seg-*EPI* magnitude and unwrapped filtered phase in the three orthogonal planes (zoomed-in red boxes, the rim is indicated with red arrows). The central vein (yellow arrows) is also clearly visible within the lesion. (B) Representative periventricular MS lesion with a paramagnetic rim. Paramagnetic rims are visible on both unwrapped phase and QSM-reconstructed images (white arrows). (For interpretation of the references to colour in this figure legend, the reader is referred to the web version of this article.)

***Paramagnetic rim lesions (PRL) -*** Recent pathology studies have demonstrated that about 30% of chronic demyelinated lesions are pathologically characterized by perilesional accumulation of iron-laden microglia and macrophages, showing evidence of smouldering demyelination and axonal loss around an inactive hypocellular core (see Fig. 4) (Frischer et al., 2015, Luchetti et al., 2018). This type of MS lesion has been defined as “chronic active/smouldering lesions”. Due to their peripheral paramagnetic iron rim, these lesions can be depicted using in-vivo susceptibility-based MRI techniques (T2*-weighted magnitude, phase images, and quantitative susceptibility mapping, QSM) at both 3 T and 7 T (Absinta et al., 2018, Absinta et al., 2016), and are therefore termed “paramagnetic rim lesions” (PRL).

Direct comparison among different MRI sequences and postprocessing techniques for PRL detection is still limited. A recent study (Huang et al., 2022) has compared QSM and high-pass-filtered (HPF) phase imaging for identifying PRL. Of 2062 MS lesions detected in 80 patients, 9.1% were identified as PRL in both QSM and HPF phase, 9.8% were PRL only in HPF phase, and the rest were rim negative. QSM-identified PRL showed stronger association with clinical disability compared to those detected by HPF phase imaging.

Overall, in vivo studies have shown that about 50% of relapsing and about 60% of progressive MS patients have at least one PRL (Absinta et al., 2019, Maggi et al., 2020). Of clinical relevance, PRL accrual has been recently linked to a more aggressive disease course and disability accumulation at a younger age and/or shorter disease duration (Absinta et al., 2019). Reasons for such association directly rely on a few typical features of these lesions: PRL are destructive (Absinta et al., 2016, Kolb et al., 2021), they do not remyelinate (Absinta et al., 2016), and they can expand over time, (Absinta et al., 2019) demyelinating the surrounding tissue and injuring axons, as corroborated by the elevation of serum neurofilament light chain in patients with PRL who are not forming new white matter lesions (Maggi et al., 2021). The recent discovery that the paramagnetic rim can significantly shrink or disappear (Absinta et al., 2021, Dal-Bianco et al., 2021) holds promise regarding its potential use as an outcome measure in clinical trials designed to halt the chronic inflammation at the lesion edge. In addition to their prognostic role, PRL appear specific to MS, as they have been rarely detected in patients with other neurological conditions (52% of MS vs 7% of non-MS in a multicenter study of 438 individuals) (Maggi et al., 2020). PRL have the promise of becoming a clinically relevant biomarker to both improve MS diagnosis and monitor treatment efficacy over time.

Overall, there are not yet imaging guidelines for the visual detection of PRL which requires specific training and remains challenging and time-consuming. The development of ML-based approaches, described in the next section, may help alleviate these issues and facilitate PRL assessment.

***Slowly evolving/expanding lesions (SELs) –*** A different computational approach, designed to detect in vivo longitudinal volumetric lesional changes not associated with gadolinium enhancement, identifies the so-called “slowly evolving/expanding lesions” or SELs. Linear and radial lesion expansion is computed as a function of the Jacobian determinant of the non-linear deformation field between baseline and follow up scans (linearity assessment requires a minimum of 3 scans) (Elliott et al., 2019). Advantages of this approach relate to the use of retrospective conventional T1-weighted and T2-weighted scans. re-analysis of the ORATORIO^a^ clinical trial found reduced rate of T1-SELs expansion in progressive patients treated with ocrelizumab vs placebo (Elliott et al., 2019). A recent study showed that SELs are independent predictors of EDSS worsening after a median follow up of 9 years (Preziosa et al., 2022). The neuropathological correlate of SELs is currently not yet determined and preliminary data showed only modest correlation with PRL (Elliott et al., 2021).

Overall, CL, PRL, and CVS have the potential to considerably improve the specificity of MS diagnosis (Junker et al., 2020, Maggi et al., 2018, Maggi et al., 2018). Moreover, studies have shown that CL, PRL, and SELs can be useful to assess prognosis (Calabrese et al., 2012, Absinta et al., 2016). Their manual assessment, however, particularly for CL, is both time-consuming and prone to inter-rater variability. As for conventional WML, some automated or semi-automated methods have been proposed to accelerate this task (Fartaria et al., 2017, Fartaria et al., 2019, La Rosa et al., 2020, La Rosa et al., 2020, Fartaria et al., 2016, Tardif et al., 2010, Barquero et al., 2020, Lou et al., 2021, Maggi et al., 2020, Dworkin et al., 2018). In the next section, we describe the challenges these approaches have been facing and how these differ from the segmentation of WML.

### 2.1 Added challenges for CL, PRL, and CVS assessment

Compared to conventional imaging biomarkers, the visual assessment of CL, PRL and CVS present some additional challenges.

**Imaging and assessment guidelines-** The first obstacle is represented by the lack of consensus guidelines for imaging protocols. Although efforts have been made to standardize the use of MRI in clinical practice for conventional biomarkers (Wattjes et al., 2021), guidelines are still in a preliminary stage for CL, PRL, and the CVS. CL were included in the MS diagnostic criteria in 2017 (Thompson et al., 2018), but, currently, there is no single gold standard sequence at 3 T for their detection in a clinical setting. PSIR, DIR, and MP2RAGE are all recommended by an international consensus (Filippi et al., 2019). However, these contrasts remain primarily acquired in research settings and are not yet widely used in clinical routine. Moreover, although 7 T MRI is increasingly used to detect CL, no guidelines have been presented yet to standardize their imaging sequences and their identification.

Regarding the CVS, in a 2016 consensus statement, the North American Imaging in MS Cooperative (NAIMS) proposed a standard radiological definition and suggested specific MRI acquisitions (Sati et al., 2016). Following these recommendations, recent studies have shown that high-resolution T2*w 3D-*EPI* or FLAIR* improve the detection of the CVS compared to clinical acquisitions (Castellaro et al., 2020, Suh et al., 2019). Nevertheless, a standardized clinical protocol for CVS detection is still missing. Among the three aforementioned biomarkers, PRL is probably the one at the earliest stages. Although recent studies support the feasibility of its assessment on clinical scans and its utility in improving the diagnosis and prognosis of MS (Maggi et al., 2020), there are currently no international guidelines for its definition nor a standardized MRI protocol for its analysis. Several different imaging modalities have been used for the PRL assessment, including phase 3D-*EPI*, susceptibility weighted imaging (SWI), QSM, and multi-echo T2* GRE at both 3 T and 7 T (Absinta et al., 2018, Absinta et al., 2016). However, there is a paucity of studies that have systematically compared the sensitivity of these acquisition techniques for PRL detection, especially when implemented at different field strengths.

These evolving or unclear criteria for CL, the CVS, and PRL, wide variety of imaging settings, and lack of clear guidelines for standardized protocols clearly jeopardize the development and wide use of these biomarkers and of targeted ML techniques.

**Expert assessment -** Even for experts, the task of segmenting CL, detecting the CVS, or classifying PRL is intrinsically more challenging than segmenting WML. CL are generally smaller in size and more affected by partial volume (PV) effects, compared to WML. The cortex is convoluted, so lesion shape is not as regular as in WM, and traditional methods of radiological evaluation (scrolling through an image stack) are less effective in this context. The detection of the CVS requires susceptibility-based MRI and its exclusion criteria need to be carefully considered when performing its assessment (Sati et al., 2016). Susceptibility-based images used to detect PRL present variability in the susceptibility signal and several artifacts, therefore experienced raters are needed. Moreover, as these three biomarkers have been so far mainly studied in research settings, clinicians do not commonly see them in clinical practice and might need specific training and dedicated time to perform a proper assessment.

### 2.2 Machine learning specific challenges

From a ML perspective, the automated segmentation or classification of CL, PRL, and the CVS faces new challenges as compared to their WML counterparts.

**Limited datasets -** An additional limitation, particularly for supervised DL-based approaches, is the scarcity and limited size of datasets in which these biomarkers were manually annotated. For their assessment, CL, CVS, and PRL all require advanced MRI sequences at high or ultra-high magnetic field and experienced raters, and this makes it difficult to have large multi-site datasets. Although national MS registries exist in most countries, the data sharing of MRI in MS is still limited and often includes only conventional sequences (Vrenken et al., 2021). Moreover, the CVS or the rim-shape in PRL are visible only on a few slices per lesion, reducing, even more, the data available to train a supervised approach.

**Inter-rater variability -** The lack of standardization for both the definition and imaging of these biomarkers contributes to a modest inter-rater variability. Barquero et al. (Barquero et al., 2020) showed that, in a cohort of 124 MS patients, approximately 38% of PRL needed a consensus review from two raters classifying PRL independently (Cohen k of 0.73). Absinta et al. observed similar inter-rater agreement between three experts at 3 T (Fleiss coefficient of 0.71), with somewhat higher intra-rater reliability (Cohen k of 0.77) (Absinta et al., 2018). Similar values were reported at 7 T for the same set of patients, whereas the agreement between 3 T and 7 T annotations was substantial (Cohen k of 0.78). In a similar way, the inter-rater agreement was shown to be moderate for the segmentation of CL (Harrison et al., 2015, Nielsen et al., 2012, Geurts et al., 2011) and high, but not perfect, for the CVS (Cohen k of 0.9) (Maggi et al., 2020, Kau et al., 2013). Imaging quality and motion artifacts are other factors to consider as they can result in inconspicuity of all three biomarkers and, therefore, contribute to poor inter-rater agreement. Overall, the inter-rater variability represents an additional challenge for the development of automated approaches, as there might be large inconsistencies in the annotations of the training or testing set due to different raters performing the manual assessment.

## 3 Methods

**Fig. 5:**
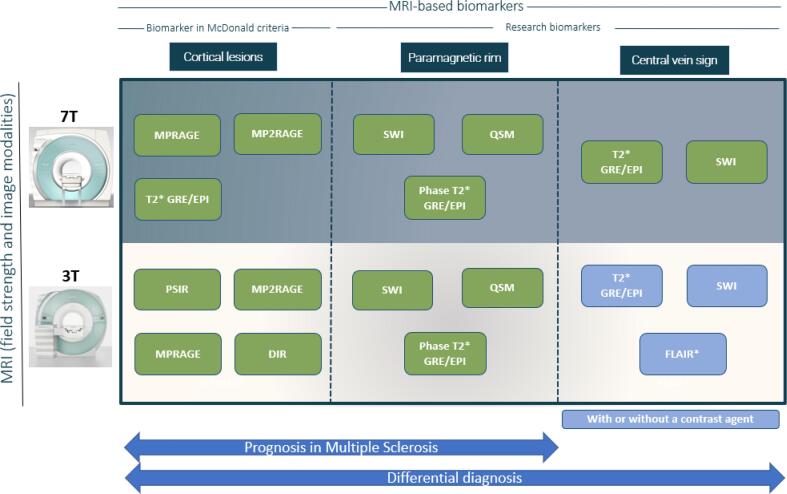
Scheme showing the main MRI sequences used for detecting each biomarker at both 3 T and 7 T.

Despite the recent discovery of the CVS and PRL and the above-mentioned challenges, a few groups have already attempted to support their analysis with automated or semi-automated ML methods. To these two novel biomarkers, we add also CL, which, although studied for several years, have only recently been assessed automatically. As there are no ML-based approaches to assess SELs yet, the prospect of analyzing this additional biomarker with ML is presented in the Discussion section. Overall, many fewer methods have been proposed for the assessment of CL, PRL, and the CVS compared to WML. In what follows, we briefly describe these state-of-the-art techniques by grouping them according to the biomarker they assess. A summary of the main characteristics for each method is presented in Table 1, and a scheme of the MRI sequences used to detect these three biomarkers at both 3 T and 7 T is shown in Fig. 5.

### 3.1 Cortical lesions

ML-based methods automatically segmenting CL have been explored with both 3 T and 7 T MRI. The first work (Tardif et al., 2010) present in the literature considered a postmortem MS brain imaged at 3 T with different sequences (T1, T2, and relative proton density) at high resolution (0.35 mm isotropic) (Tardif et al. (2012)). Tardif et al. (Tardif et al., 2010) proposed to first identify the cortical and white matter surfaces, then extract laminar profiles between the two tissues, and finally apply a k-means classifier to the profile intensity and shape features to parcellate the cortex and detect lesions. Although showing promising results on one postmortem MS brain, this method was never validated with larger cohorts nor in-vivo data. A few years later, Fartaria et al. (Fartaria et al., 2016) proposed the first automated method for the segmentation of both WM and cortical lesions. In their study, they analyzed a cohort of 39 early-stage MS patients and considered both conventional (FLAIR, MPRAGE) and advanced (MP2RAGE, DIR) MRI sequences at 3 T. In a nutshell, their method consisted of co-registering the different MRI contrasts, leveraging prior tissue probability maps from existing brain atlases of healthy subjects, and finally classifying each voxel either as being a lesion or healthy tissue with a k-nearest neighbor (k-NN) algorithm. Additionally, as post-processing, all lesions smaller than 3.6 µL were discarded, and a region-growing algorithm was applied to improve the lesion delineation. Results were promising, showing a CL detection rate of 62% when advanced imaging (FLAIR, MP2RAGE, and DIR) was included. An extension of this segmentation framework with a Bayesian partial volume (PV) estimation method was presented by the same authors (Fartaria et al., 2017). They argued that CL, being generally small and located at the interface between WM and GM, suffer from strong PV effects. The addition of this PV model indeed improved the delineation of CL in terms of both total lesion volume estimation and dice coefficient (Fartaria et al., 2017).

The same research group also proposed a different segmentation method for WML and CL using only 7 T MP2RAGE images (called MSLAST: Multiple Sclerosis Lesion Analysis at Seven Tesla) (Fartaria et al., 2019). MSLAST computes tissue concentration maps with a PV algorithm and unifies them based on topological constraints. A connected-components analysis is then performed on gray matter and cerebrospinal fluid maps, and small components are classified as MS lesions. This method was evaluated with 25 MS patients’ scans from two research centers and reached a 58% patient-wise CL detection rate (when 6 μL was considered as minimum lesion volume) with a false positive rate of 40%. Moreover, it showed promising scan-rescan repeatability within the same session, with a mean total lesion volume difference (WML and CL combined) of 0.29 mL (mean total lesion volume 5.52 mL), vs 0.13 mL for the manual segmentations. More recently, DL-based approaches have been presented as well (La Rosa et al., 2020, La Rosa et al., 2020). In the first study, La Rosa et al. proposed a framework for the automated segmentation of WML and CL at 3 T using FLAIR and MP2RAGE (La Rosa et al., 2020). Their method extracts 3D patches of 88x88x88 voxels from the two MRI contrasts and feeds them to a convolutional neural network (CNN). The CNN, inspired by the U-Net, has an encoder and decoder path, each one with three resolution levels. Evaluated on two datasets acquired in different centers, for a total of 90 MS patients, the framework showed competitive performance, with a CL detection rate of 76% and a false positive rate of 29%.

**Fig. 6:**
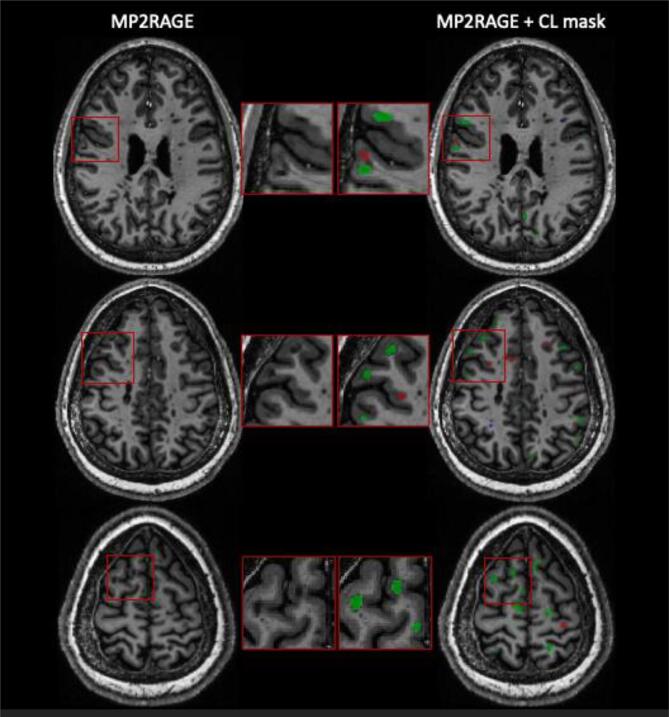
Three representative axial slices from one MS patient showing the CL segmentation results of an automated CL segmentation method (La Rosa et al., 2020). 7 T MP2RAGE (left column) and CL mask (right column) showing true positives (green), false negatives (red), and false positives (blue) of the automated approach with respect to the CL manual segmentation. (For interpretation of the references to colour in this figure legend, the reader is referred to the web version of this article.)

In a second study, the same group proposed a similar approach, this time tailored exclusively for the detection of CL using multi-contrast 7 T MRI (La Rosa et al., 2020). The contrasts considered were MP2RAGE, T2*-weighted GRE, and T2*-w 3D-*EPI*. A cohort of 60 patients was analyzed with a total of over 2000CL manually segmented by two experts. The CNN architecture proposed was similar to the one just described, but with a modified output. In addition to the CL segmentation, the CNN provided a classification into two types (leukocortical and intracortical/subpial lesions) and a separate branch with a simple tissue segmentation in WM/GM. CL were correctly classified into the two types by the network with an accuracy of 86%. Setting a minimum lesion size of 0.75 μL, it achieved a CL detection rate of 67% with, however, a quite high false positive rate of 42% (see Fig. 6). Importantly, about 24% of these false positives were retrospectively judged as CL or possible CL by an expert (La Rosa et al., 2020).

In a following publication (La Rosa et al., 2022), this method was further improved and evaluated on a multi-site dataset. Its main modifications included an added resolution level in the CNN architecture, a larger 3D patch input size of 96x96x96 voxels, and the use of the focal loss for training. Finally, a domain adaptation approach was applied to verify the performance on external datasets. On 20 MRI scans of patients imaged in a different center, this method achieved superior performance (CL detection rate of 71%) compared to MSLAST (48%) when setting a minimum lesion size of 6 μL.

### 3.2 The central vein sign

As of today, two automated ML methods for the classification of MS lesions as CVS+ (MS lesions showing the presence of the CVS) or CVS- (MS lesions without the CVS) have been proposed in the literature (Maggi et al., 2020, Dworkin et al., 2018). Both approaches were developed and evaluated only with 3 T MRI. Dworkin et al. (Dworkin et al., 2018) proposed a probabilistic method based on the Frangi vesselness filter (Frangi et al., 1496). They first perform an automated WML segmentation using T1 and FLAIR 3D MRI volumes acquired at 3 T (Valcarcel et al., 2018, Valcarcel et al., 2018) and obtain a map of the veins by applying the vesselness filter to a T2*w 3D *EPI* image. Confluent lesions are then separated, and lesion centers are detected by textural analysis (Dworkin et al., 2019). Periventricular lesions are discarded as suggested by consensus guidelines (Sati et al., 2016), and a permutation algorithm is applied to verify whether veins occur at the lesions’ centers more often than would be expected due to random chance. Finally, to account for scan motion, the single lesion CVS + probabilities are weighted by the noise in their T2*-w 3D-*EPI* intensities and averaging across the total number of lesions, a patient-wise CVS value is obtained. This method was evaluated on a cohort of 31 adults, of whom 16 had MS. When considering a 40% cutoff rule, the method yielded a sensitivity of 0.94 and a specificity of 0.67 on a patient-wise classification level. The performance of the method on a lesion-wise level was not assessed. Although still far from experts’ performance, this was a first attempt to automatize the CVS assessment and encouraged further improvements.

Maggi, Fartaria et al. (Maggi et al., 2020) introduced an optimized CNN for the automated CVS assessment, called CVSnet. CVSnet is inspired by the VGGnet (Simonyan and Zisserman, 2015) but composed of only three convolutional layers followed by rectified linear unit (ReLU) activations. Dropout was applied in each layer, and then two fully-connected layers of size 32 and 2, respectively, were added to provide the output. The authors selected 3D patches of size 21x21x21 voxels as input for the network, where each patch was centered on an MS lesion and FLAIR* was the only MRI contrast used. Moreover, an ensemble of 10 networks with the same architecture was trained and the probability outputs were averaged to provide the final prediction. This study considered a cohort of 80 patients imaged at three different sites, of whom 42 had MS, 35 an MS-mimic, and 5 an unknown diagnosis. On the test set, CVSnet reached a lesion-wise sensitivity, specificity, and accuracy of 0.83, 0.75, and 0.79, respectively. On a patient-wise level, using a 50% cut-off, CVSnet achieved a sensitivity, specificity, and accuracy of 0.89, 0.92, and 0.90, respectively, outperforming the vesselness filter (Frangi et al., 1496) and approaching expert performance. However, as argued by the authors, these results are not directly comparable with those of Dworkin at al. (Dworkin et al., 2018), as the CVSnet considered different exclusion criteria to pre/select the lesions, and the initial lesion segmentation was performed manually.

### 3.3 Paramagnetic rim lesions

To our knowledge, only three methods have been proposed so far for the detection of rim-like features and classification of PRL (Barquero et al., 2020, Lou et al., 2021, Zhang et al., 2022). All three methods considered 3 T MRI sequences, whereas 7 T imaging has not yet been explored for the automated assessment of PRL. Barquero et al. (2020) introduced a DL-based approach (called RimNet) for the semi-automated classification of PRL, which considered 3D FLAIR and T2*w 3D-*EPI* and phase 3D-*EPI* images. RimNet’s architecture is inspired by the VGGnet (Simonyan and Zisserman, 2015) and composed of two parallel CNN (one for either FLAIR or T2*w 3D-*EPI* image and one for the phase 3D-*EPI* image), where each CNN is made of three convolutional layers followed by a max-pooling operation. 3D patches of size 28x28x28 (centered around each MS lesion) are fed to each branch, and both high-level and low-level feature maps are concatenated. An automated lesion segmentation based on FLAIR and MPRAGE/MP2RAGE (La Rosa et al., 2020, La Rosa et al., 2019) was modified by an expert to split confluent lesions. The performance of RimNet was assessed on a cohort of 124 adults with MS who underwent 3 T MRI at two different sites with two scanners from the same vendor. Two experts annotated PRL independently and reached consensus in a joint session (462 PRL in total). The proposed multimodal approach based on FLAIR and phase 3D-*EPI* image achieves lesion-wise sensitivity and specificity of 0.70 and 0.95, respectively. When considering a previously identified clinical threshold of 4 PRL (Oladosu et al., 2021) for classifying patients as “chronic active” and “non-chronic active”, RimNet reaches an accuracy of 0.90 and an F1-score of 0.84. These values are within 5% of the single experts’ metrics, suggesting that RimNet could be a valuable tool in supporting the PRL analysis. The main drawback of RimNet, however, is that the method is not fully automated, as confluent lesions were split manually by an expert.

Lou et al. (Lou et al., 2021), on the other hand, proposed a fully automated ML method for PRL assessment. They considered a cohort of 20 subjects with MS imaged with 3D FLAIR, 3D MPRAGE, and T2*-w 3D-*EPI* and phase 3D-*EPI* images. One neurologist inspected the T2* magnitude and unwrapped phase images and annotated PRL (113 PRL over the entire cohort). The automated pipeline, after some pre-processing steps that included lesion segmentation (Valcarcel et al., 2018, Valcarcel et al., 2018), lesion center detection (Dworkin et al., 2019), and lesion labeling, consisted of extracting 44 different lesion-wise radiomic features. A random forest classifier was then fitted on these features, and its ability to classify PRL was evaluated on a test set of 4 patients. Sensitivity and specificity of 0.75 and 0.81, respectively, were achieved. Although fully automated, this study has three limitations. First, the extremely small testing dataset (4 patients only with 47 PRL), annotated by a single expert, does not guarantee the generalization of the proposed method. Second, all patients analyzed had at least one PRL, and this might add a bias to the trained model. Finally, as acknowledged by the authors, about 65% of misclassified lesions were confluent, highlighting the need for a better solution to address these lesions.

Inspired by these two previous works, Zhang et al. introduced QSM-RimNet (Zhang et al., 2022), a QSM-based approach that combines a two-branch feature extraction network and a synthetic minority oversampling technique. QSM-RimNet receives as input 3D patches of size 32x32x16 voxels where a masking out of non-lesional area is applied. One branch of the network employs residual blocks to extract convolutional features from QSM and FLAIR images, whereas the second consists in a fully-connected network that processes previously obtained radiomic features. Convolutional and radiomic features are concatenated and a minority oversampling network is used to alleviate the issue of class imbalance. Finally, a probability of being a PRL is assigned to each lesion. QSM-RimNet was evaluated with a stratified 5-folds cross-validation over 172 MS patients with a total of 177 PRL. Compared to RimNet and the automated approach of Lou et al., it outperformed both methods achieving a lesion-wise sensitivity and specificity of 0.68 and 0.99, respectively, although the differences were not statistically significant. Ablation studies showed that fusing convolutional and radiomic features improves the PRL identification (Zhang et al., 2022). Of note, QSM-Rimnet is not fully-automated as during training and evaluation it relies on manual corrections by experts of both PRL and confluent lesions. Similarly to RimNet, this strong limitation currently prevents its wider deployment and applicability.

Overall, two methods have tackled the PRL detection problem considering mainly the T2*-w 3D-*EPI* sequence and one method has focused on the QSM. Thus, none of the three frameworks has investigated the effect of differences in SWI and QSM processing on ML-based tools performance and this important aspect should be explored in future studies.

## 4 Discussion

The methods described in the present review tackle challenging and clinically relevant problems. Automated and reliable solutions for detecting, classifying, and segmenting CL, PRL, and CVS are needed to improve the standardization of these biomarkers and facilitate their assessment in clinical routine. As of today, however, these methods are still in an early stage and are slightly less sensitive than WML segmentation approaches.

Nevertheless, such tools would provide obvious advantages, either as stand-alone or adjunctive approaches as all three biomarkers are difficult and time-consuming to analyze using conventional radiological workflows. In these particular cases, manual reading is so involved that automated methods might actually boost the biomarkers' widespread adoption. First, they can substantially reduce analysis time, as compared to a manual rating. Maggi, Fartaria et al., for instance, showed that CVSNet was 600-fold faster on the test set compared to the manual assessment (4 s vs 40 min) when considering a 50% CVS + lesions criteria to distinguish MS from MS mimics (Maggi et al., 2020). A lower time gain, however, would be expected if CVS + lesion-count criteria, such as the 3-lesion and 6-lesion, were to be considered. Reduced analysis time can be predicted also for PRL and CL assessment. In La Rosa et al. (La Rosa et al., 2020), for instance, the automated CL segmentation of one subject is computed on average in 20 s. Although a direct comparison with the manual labeling was not reported, segmenting CL manually is known to be a much more time-consuming process. A second main advantage of automated ML methods is their ability to base their decision on 3D multi-contrast MRI analyzed simultaneously. This stands in contrast to expert reviews, which typically involve comparison of 2D slices across several contrast mechanisms in a variety of planes and are thus inherently limited in the amount of information that can be readily gleaned.

### 4.1 Common trends

Some common trends can be observed in most of the proposed pipelines. The large majority of the methods are supervised, relying on expert annotations. Regarding the DL-based approaches, they all used patch-based 3D CNN, exploiting the 3D intrinsic information, and often considered more than one MRI contrast simultaneously. In addition, a shared tendency consists of the use of relatively shallow architectures, with a limited number of trainable parameters, due to the lack of large datasets (La Rosa et al., 2020, La Rosa et al., 2020, Barquero et al., 2020, Maggi et al., 2020). Combining this with extensive data augmentation techniques can help when datasets are small and unbalanced. Alternatively, other groups have tackled the problem of overfitting by proposing approaches based on classical ML techniques, such as k-NN (Fartaria et al., 2017, Fartaria et al., 2016) or random forest classifier (Commowick et al., 2018). In these studies, either intensity-based, radiomic, or probabilistic features are extracted and then fed to the respective classifier. Overall, their current performance is inferior compared to their DL-based counterparts.

In addition, some common pre-processing steps can also be identified. First, some methods use intensity normalization techniques, either based on entire 3D volumes (Lou et al., 2021, Dworkin et al., 2018, Fartaria et al., 2017, Fartaria et al., 2019, La Rosa et al., 2020, La Rosa et al., 2020) or on single patches (Barquero et al., 2020, Maggi et al., 2020). Second, the approaches using multiple MRI contrasts always register all images to the same space (Lou et al., 2021, Fartaria et al., 2017, Fartaria et al., 2019, La Rosa et al., 2020, La Rosa et al., 2020). Registration errors might affect the methods’ performance. Finally, a shared pre-processing step in all approaches for the CVS or PRL assessment is the prior WML segmentation, obtained either manually (Maggi et al., 2020) or with an automated tool (Barquero et al., 2020, Lou et al., 2021, Dworkin et al., 2018). In both cases, this can be a source of error that negatively affects the subsequent biomarkers’ classification accuracy.

### 4.2 Current limitations

Currently, a major limitation hinders the deployment of the above-described methods to the clinic: the methods proposed were trained and evaluated on small datasets acquired from one or at most two centers. Moreover, the MRI protocols used were often similar and not representative of the current diversity of images acquired in the clinics, including different processing, scans affected by noise and artifacts or protocols missing certain modalities. Therefore, the automated ML methods’ robustness on larger datasets and different scanners, especially from multiple vendors, remains to be proven. This limitation is emphasized by the current lack of standardized acquisition protocols which increases the diversity of the MRI sequences considered for the same biomarkers. This also represents a major hurdle for potential regulatory approval of such methods. As regulatory approval is necessary for widespread adoption in the clinics, which is, in turn, the prerequisite for the availability of large datasets, this is currently a circular dependency issue.

In addition, the achieved performance levels of the automated ML methods are still inferior compared to the human experts. Considering the high inter-rater variability and the limited amount of data available, there is also a considerable risk of having methods that perform well on data annotated by a single expert and not as well with annotations from other raters. To mitigate this issue, several methods have already considered consensus annotations from two or more experts (La Rosa et al., 2020, Barquero et al., 2020, Maggi et al., 2020). Regarding CL, no automated method presented in the literature was compared, on the same dataset, with the experts’ inter-rater variability, thus a proper evaluation is not possible. With respect to CVS, Maggi, Fartaria et al. (Maggi et al., 2020) compared the performance of CVSnet with the consensus of two experts. Following the “50% rule,” CVSnet achieved on the testing set a classification accuracy of 79%, whereas the experts reached 100% accuracy in differentiating MS and mimic diseases. In a similar way, Barquero et al. (Barquero et al., 2020) compared RimNet’s performance with those of two experts in classifying PRL. In a lesion-wise analysis, RimNet achieved a sensitivity of 71% and a negative predictive value of 96%, approaching the experts, who reached 78% and 98%, respectively.

Another main limitation is represented by the fact that some methods presented are not fully automated. CVSnet (Maggi et al., 2020), for instance, used manually annotated MS lesion masks in which lesions were excluded based on the NAIMS criteria (Sati et al., 2016), whereas in the pipeline proposed by Dworkin et al. (Dworkin et al., 2018), scans affected by noise were discarded following a manual rating. Similarly, RimNet (Barquero et al., 2020) exploits lesion masks where confluent lesions have been previously split into single units by an expert. In contrast, all methods described to date for CL segmentation or detection are fully automated (Fartaria et al., 2017, La Rosa et al., 2020, La Rosa et al., 2020, Fartaria et al., 2016). Another persistent issue in the automated analysis of the CVS and PRL is the presence of confluent lesions. Large, periventricular white matter lesions which include several single units pose additional challenges as the current methods classify each lesion singularly (Lou et al., 2021, Dworkin et al., 2018), and some of them extract 3D patches centered on the lesion of interest (Barquero et al., 2020, Maggi et al., 2020). In RimNet (Barquero et al., 2020), for instance, an expert manually split confluent lesions, whereas Lou et al. observed a consistent drop in performance in PRL classification in the presence of confluent lesions (Lou et al., 2021). Although methods to automatically split confluent lesions have been proposed (Dworkin et al., 2019, Zhang et al., 2021), further developments are needed in order to properly apply these in the presence of the CVS or PRL.

Finally, for every automated tool the regulatory environment remains a critical barrier, as up to date less than 90 AI/ML-based medical devices or algorithms have been approved by the US Food & Drugs Administration (FDA). This challenge, however, is not unique to the three biomarkers considered (Pinto et al., 2020) but shared also by automated approaches segmenting WML or estimating brain atrophy.

### 4.3 Future research avenues

**Standardization of the biomarkers’ assessment-** The first two necessary steps toward the improvement of the above-referred approaches are the validation of the biomarkers’ specific criteria and standardization of the relative MRI protocols. CL have been recently included in the MS diagnostic criteria (Thompson et al., 2018), however, a consensus on imaging and on their definition is still missing. In a similar way, PRL urgently need a consensus definition and standardized clinical protocols, whereas the initial criteria proposed for the CVS (Sati et al., 2016) need to be updated in light of the latest studies. This would clarify the automated methods’ goals, which so far have been extremely dependent on specific expert labeling of each dataset or on the specific criteria adopted.

**Standardization and extensive validation of the automated methods** - Currently, it is difficult to compare the performance of automated ML methods considering different criteria (such as the minimum lesion size) and being evaluated on private datasets. In the future, the generalization of the proposed methods should be validated on large, multi-site datasets with standardized metrics. For this purpose, we urge research groups to organize grand challenges and release publicly available datasets with manual annotations of CL, PRL, and CVS. As already proved for several other tasks in medical imaging (Antonelli et al., 2021), including for WML segmentation (Carass et al., 2017, Commowick et al., 2018), such open data initiatives boost on the one hand the development of state-of-the-art methods, and on the other hand, help set benchmarks for a fair assessment. Only 5 of the 12 methods covered in this review are publicly available. In order to extend their usage and foster a culture of open science, research groups should make their code publicly available and possibly provide Docker (Docker, 2014)/Singularity (Kurtzer et al., 2017) images to facilitate their evaluation. Moreover, as successfully done for WML segmentation (Valverde et al., 2019), domain-adaptation techniques should also be explored in order to improve robustness of the automated ML methods to noise, artifacts, and different protocols. So far, all three biomarkers have been primarily studied at 3 T and 7 T, and therefore robust methods able to work with images acquired at both magnetic field strengths would be very valuable. Machine learning algorithms could exploit 7 T enhanced spatial resolution and tissue contrast by domain adaptation techniques to improve their performance on 3 T imaging, which will continue to be the main tool for clinicians as well as for clinical research and trials for the foreseeable future. Although it would be highly desirable to have methods that work also at the most accessible field strength of 1.5 T, this seems currently unlikely as the sensitivity to these biomarkers is field-dependent.

**Transfer learning -** Considering the scarcity of large, annotated datasets, an additional strategy that should be explored consists of transfer learning. Sharing of neural network weights between research groups could foster interdisciplinary applicability of CNN trained on relatively large datasets towards different purposes, such as CL, PRL, and CVS, by fine-tuning the trained models in smaller datasets. Potential advantages would include a shorter training time and robust feature extraction across different MRI device manufacturers or different pulse sequence acquisition parameters (Valverde et al., 2021).

**Longitudinal assessment -** Another possible research direction is an expansion of the current methods to analyze longitudinal data. To the best of our knowledge, only one study has tackled the automated longitudinal assessment of CL at 3 T (Fartaria et al., 2019), whereas PRL evolution over time has not yet been assessed with automated approaches. CL are known to play a major role in disease progression (Mainero et al., 2015) and considerable changes in their volume were observed in longitudinal studies (Calabrese et al., 2008, Faizy et al., 2019). Of similar interest, PRL and slowly-evolving lesions (SELs) volume assessment over time is a plausible future clinical measure of treatment response (Absinta et al., 2021, Dal-Bianco et al., 2021, Elliott et al., 2019, Elliott et al., 2019). Therefore, automated longitudinal assessment of both CL and PRL could be of high relevance. Regarding SELs, longitudinal WML segmentation approaches (Lladó et al., 2012) could be adapted to track their evolution in a fully-automated way. This would facilitate their assessment as currently, following an automated cross-sectional WML segmentation, the lesion masks at each timepoint are manually reviewed (Elliott et al., 2019).

**Joint assessment of multiple biomarkers-** To date, all the methods proposed tackled the assessment of a single lesional biomarker, although in the case of CL some methods consider WML as well (Fartaria et al., 2019, La Rosa et al., 2020, Fartaria et al., 2016). Future work may aim at automatically analyzing multiple biomarkers in a unified framework (eg. with the same input images and algorithm) as this would be extremely useful for research purposes or in clinics. Moreover, ML-based algorithms have the potential to be useful also for prediction purposes. A few automated methods based either on MRI (Tousignant et al., 2021, Marzullo et al., 2019, Roca et al., 2020), optical coherence tomography (Montolío et al., 2021), or clinical information (Pinto et al., 2020) have already been presented to predict MS progression. Specifically to the biomarkers considered in the present review, Treaba et al. have proposed a ML approach for the regression of both CL and PRL, in the same cohort of patients, with disability progression (Treaba et al., 2021;3(3):fcab134.). In this prospective, longitudinal study, the authors analyzed brain scans of 100 MS patients using 7 T susceptibility-sensitive MRI in which CL and PRL were segmented manually. Although the study had some limitations, including the fact that the disability progression was assessed only by the EDSS and only one ML-based method (gradient boosting algorithm, XGBoost) was tested, it showed that 7 T MRI and the combination of different biomarkers are promising in predicting MS disability progression. Future studies should aim to combine the automated assessment of multiple biomarkers with clinical information and other relevant markers to predict clinical outcomes or treatment effect.

**Explainable AI -** As discussed in this paper, ML methods combined with specialized MRI sequences could play a fundamental role in supporting the diagnosis of, and prognostication in, MS. However, the complexity of DL algorithms hinders their interpretation, which has led some to consider these methods as “black boxes.” The lack of an obvious connection between biology, pathophysiology, and features revealed by DL might diminish clinicians’ confidence in these algorithms, again hindering the adoption of such tools in clinical research and healthcare. Explainable AI (XAI) methods are needed as to on one side provide uncertainty estimates regarding the output provided and on the other side transparency on the decisions taken by the DL-models. By explainability, we refer to a set of domain features such as pixels of an image or human-understandable high-level attributes that contribute to the output decision of the model and its internal working. To our knowledge, there are only two groups that have investigated XAI in MS. Eitel et al. (Eitel et al., 2019) explored explainability to reveal relevant voxel-wise locations that a trained CNN uses for distinguishing between a normal and MS brain MRI. They found that diagnostic success relied on the appearance of both lesions and non-lesional tissue (thalamus). Nair et al.Nair et al. (2020) studied the uncertainty of DL-based lesion segmentation to quantify the AI model reliability. Interestingly, their results showed that discarding lesions with high estimated uncertainty from the output segmentation would improve the performance of the model. These two pioneering approaches strengthen the idea that explainability and uncertainty measures can reliably provide new insights into how DL models for MS work and potentially improve them and increase their transparency.

Overall, we believe that developing explainable AI tools is crucial in the ML MS research roadmap and would have an impact at both methodological and clinical levels. First, explainable DL in MS would provide new insights into model decisions and help identify any bias. Second, the inclusion of uncertainty and explainability will help in increasing the confidence of clinicians considering their use, as well as improve the quality of decision making and ultimately the clinical impact. Finally, they may foster a better understanding of MS progression by generating biologically interpretable measures of inflammation and degeneration.

## 5 Conclusions

To summarize, automated or semi-automated ML-based approaches aiming to segment and classify CL, CVS, and PRL are still in an early stage. Nevertheless, these pioneering methods have the potential to provide standardized identification of the biomarkers and facilitate their large-scale assessment in clinical routines. Automated or semi-automated tools could considerably reduce the current amount of time and effort needed for a manual assessment. To date, however, some limitations still hinder a broader adoption of these tools. First, there is a general need for consensus criteria and standardized clinical protocols for all three aforementioned biomarkers. Further, a major barrier to the automated methods’ deployment is their lack of validation on multi-center datasets acquired with different protocols. Future work should focus on improving the robustness of the automated methods, extending their framework with longitudinal data, and including interpretable measures into their decisions. Finally, we encourage research groups to organize grand challenges and release publicly available datasets. This would boost the development of new methods and provide benchmarks for a fair and standardized comparison that is currently lacking.

## CRediT authorship contribution statement

**Francesco La Rosa:** Conceptualization, Methodology, Validation, Formal analysis, Investigation, Data curation, Writing – original draft, Writing – review & editing, Visualization. **Maxence Wynen:** Methodology, Visualization, Formal analysis, Investigation, Data curation, Writing – original draft, Writing – review & editing. **Omar Al-Louzi:** Methodology, Validation, Formal analysis, Data curation, Writing – original draft, Writing – review & editing. **Erin S Beck:** Methodology, Validation, Resources, Data curation, Writing – original draft, Writing – review & editing, Visualization. **Till Huelnhagen:** Resources, Writing – original draft, Writing – review & editing, Visualization. **Pietro Maggi:** Resources, Writing – original draft, Writing – review & editing, Visualization. **Jean-Philippe Thiran:** Investigation, Supervision, Project administration, Funding acquisition. **Tobias Kober:** Investigation, Writing – original draft, Writing – review & editing. **Russell T Shinohara:** Validation, Investigation, Writing – original draft, Writing – review & editing. **Pascal Sati:** Validation, Investigation, Writing – original draft, Writing – review & editing. **Daniel S Reich:** Validation, Formal analysis, Investigation, Resources, Writing – original draft, Writing – review & editing. **Cristina Granziera:** Validation, Resources, Writing – original draft. **Martina Absinta:** Validation, Formal analysis, Investigation, Resources, Data curation, Writing – original draft, Writing – review & editing, Visualization. **Meritxell Bach Cuadra:** Conceptualization, Methodology, Validation, Investigation, Resources, Writing – original draft, Writing – review & editing, Supervision, Project administration, Funding acquisition.

## Declaration of Competing Interest

The University Hospital Basel (USB), as the employer of C.G., has received the following fees which were used exclusively for research support: (i) advisory board and consultancy fees from Actelion, Genzyme-Sanofi, Novartis, GeNeuro and Roche; (ii) speaker feesfrom Genzyme-Sanofi, Novartis, GeNeuro and Roche; (iii) research support from Siemens, GeNeuro, Roche. M.A. has received consultancy fees from GSK and Sanofi-Genzyme. P.M. has received support from Biogen and Cliniques universitaires Saint-Luc Fonds de Recherche Clinique. D.S.R. has received research support from Abata, Sanofi-Genzyme, and Vertex. The other authors have no known competing financial interests or personal relationships that could have appeared to influence the work reported in this paper.

## Data Availability

No data was used for the research described in the article.

## Footnotes

a A phase 3, randomized, parallel-group, double-blind, placebo-controlled trial.
