# Supplementary material for: Cortical lesions, central vein sign, and paramagnetic rim lesions in multiple sclerosis: Emerging machine learning techniques and future avenues
Source: Neuroimage Clin. 2022 Sep 24;36:103205. doi: 10.1016/j.nicl.2022.103205 (PMC9668629; doi:10.1016/j.nicl.2022.103205)
Supplement: Supplementary data 1 [file mmc1.docx]

Supplementary material


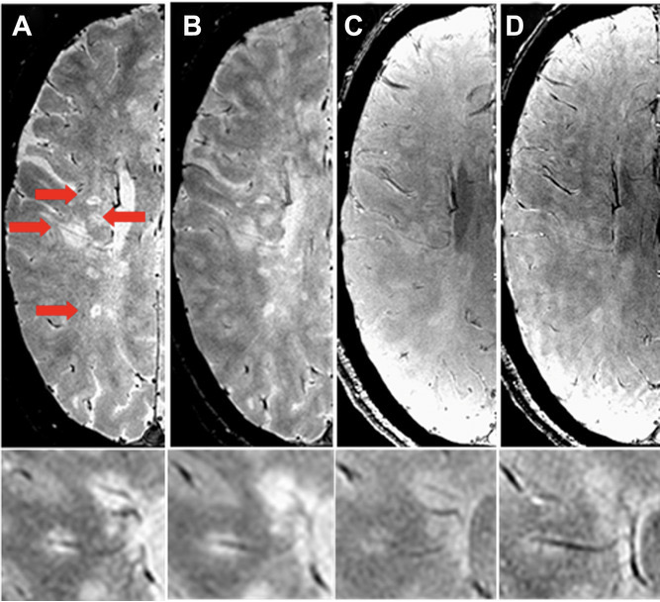


Supplementary material, Figure 1: From left to right: (A) T2* with high EPI; (B) standard T2* Philips; (C) SWAN imaging; (D) standard T2* GE. The red arrows point to the CVS visible in the T2* with high EPI. The top and bottom row show magnified views of two different patients. Figure reprinted with permission from Samaraweera et al., The Central Vein Sign in Multiple Sclerosis Lesions Is Present Irrespective of the T2* Sequence at 3 T, Journal of Neuroimaging (2012).
